# Ankylosaur Remains from the Early Cretaceous (Valanginian) of Northwestern Germany

**DOI:** 10.1371/journal.pone.0060571

**Published:** 2013-04-03

**Authors:** Sven Sachs, Jahn J. Hornung

**Affiliations:** 1 Engelskirchen, Germany; 2 Geowissenschaftliches Zentrum, Georg-August-Universität, Göttingen, Germany; Royal Ontario Museum, Canada

## Abstract

A fragmentary cervico-pectoral lateral spine and partial humerus of an ankylosaur from the Early Cretaceous (early Valanginian) of Gronau in Westfalen, northwestern Germany, are described. The spine shows closest morphological similarities to the characteristic cervical and pectoral spines of *Hylaeosaurus armatus* from the late Valanginian of England. An extensive comparison of distal humeri among thyreophoran dinosaurs supports systematic differences in the morphology of the distal condyli between Ankylosauria and Stegosauria and a referral of the Gronau specimen to the former. The humerus fragment indicates a rather small individual, probably in the size range of *H. armatus*, and both specimens are determined herein as ?*Hylaeosaurus* sp.. A short overview of other purported ankylosaur material from the Berriasian-Valanginian of northwest Germany shows that, aside from the material described herein, only tracks can be attributed to this clade with confidence at present.

## Introduction

**Figure 1:**
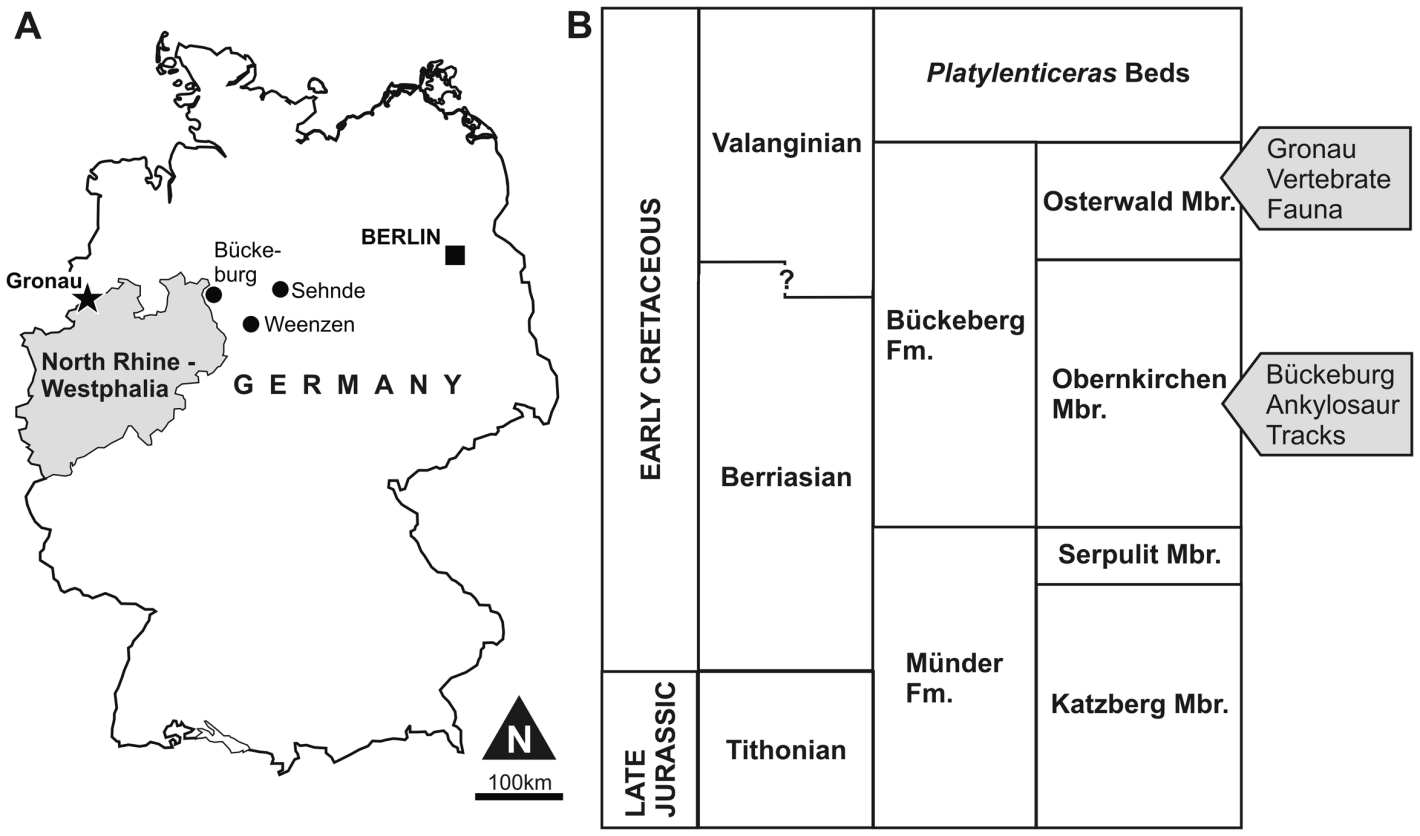
Geographic and stratigraphic information. **A**, Location map. **B**, Lithostratigraphy of the basal Cretaceous in northwestern Germany (after [15], [17], [41]; simplified). The stratigraphic positions of the Gronau vertebrate site and the ankylosaur tracks from Bückeburg are indicated.

The Gerdemann clay-pit in Gronau in Westfalen (northwestern Germany, Fig. 1a) has yielded a considerable amount of vertebrate fossils, including remains of fishes, turtles, plesiosaurs, crocodilians and dinosaurs [1], [2], [3], [4], [5]. The fossils were collected mainly during the first decades of the 20th century and sold or donated by the clay-pit owner to various museums and collections in northern Germany and the Netherlands. Most specimens are kept in the Geomuseum of the University of Münster and in the Museum TwentseWelle in Enschede.

**Figure 2:**
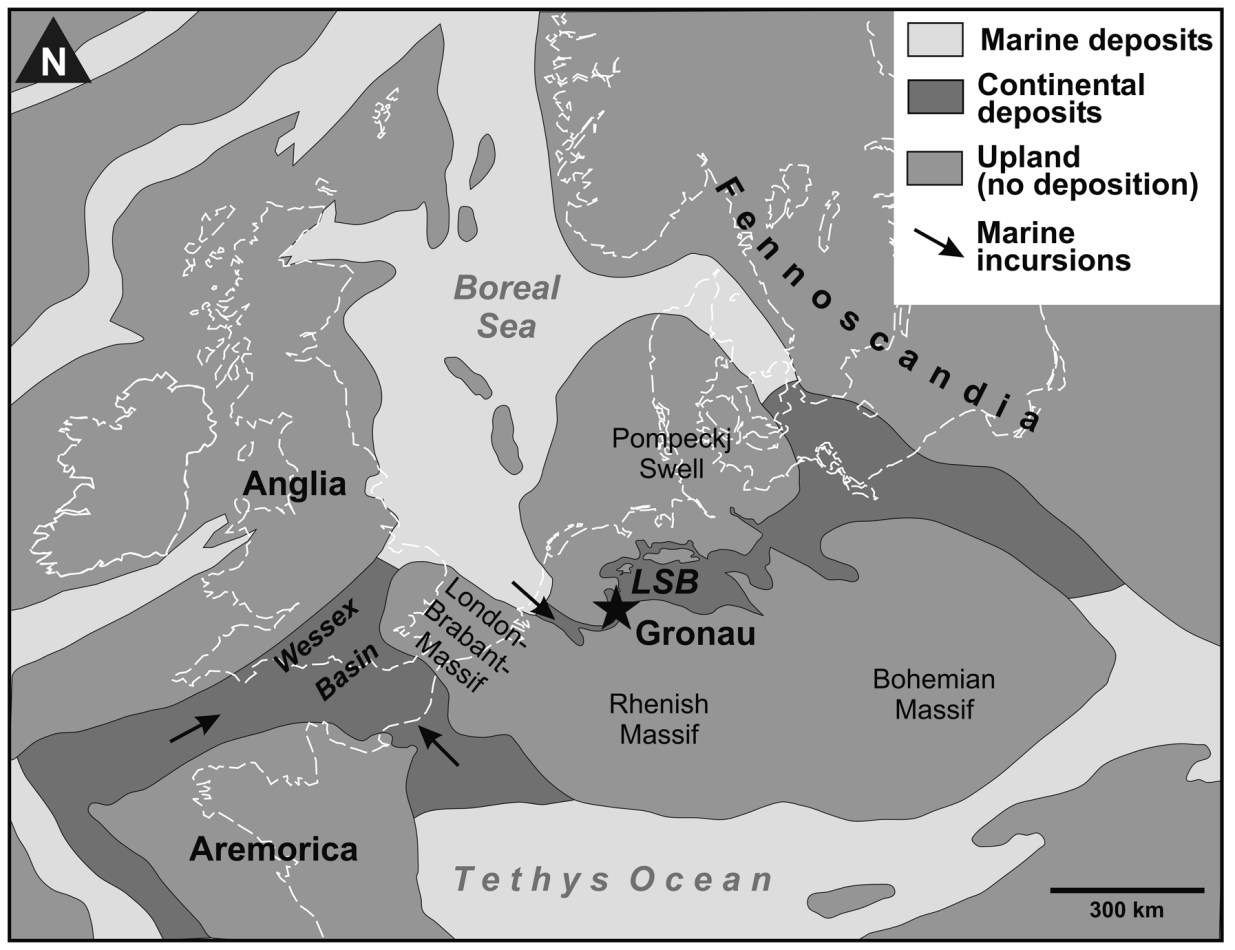
Paleogeography of the Berriasian/early Valanginian of central Europe (after [**41**]; modified). The location of the Gronau vertebrate site is indicated.

The exposed strata in Gronau belong to the argillaceous facies of the upper Bückeberg Formation (Osterwald Member, Fig. 1b). Mostly deposited under limnic conditions in the Early Cretaceous Lower Saxony Basin. The western part of the basin, including the Gronau region, occasionally came under brackish to marine influence via passages to the Boreal Sea ([6]; Fig. 2). These brackish and marine intervals increase in number and prominence in the youngest part of the succession and may correlate with diversity peaks in the aquatic fossil faunas. However, according to paleogeographical reconstructions, the Gronau area was not in immediate proximity to shorelines (distance ca. 5–10 km [7]) and the mixture of aquatic (fishes, plesiosaurs, some turtles), semiaquatic (crocodilians) and terrestrial (dinosaurs) vertebrates in the rather monotonous pelitic succession is remarkable. Possibly subaqueous density flows have played a role in the formation of this taphocoenosis by transporting carcasses from the littoral zone deeper into the basin [5].

Here we describe ankylosaur remains from the Gerdemann clay-pit. The material includes a humerus fragment [4] and a cervico-pectoral spine that was recently discovered by the authors in the collection of the Drilandmuseum in Gronau. A short overview of the thyreophoran record of the Early Cretaceous of Germany is also provided.

## Materials and Methods

In the context of this paper, “spines” refer to slender, pointed osteoderms of ankylosaurs, whereas “spike” is used for the similarly shaped parascapular/parasacral and caudal elements in stegosaurs. Positional terminology for osteoderms used herein follows Scheyer and Sander [8]. Surfaces and features of elements closer to the epidermis are referred to as “external”. Surfaces and features closer to the body wall (towards the body axis) are referred to as “basal”. Pointed structures terminate externally in an “apex”. We refer to the most ventrolateral line of osteoderms, which often point more or less laterally to the body axis, as “lateral osteoderms”, whereas elements dorsal and medial to this row are considered as “dorsal osteoderms”. The lateral dermal armor may be organized in subsections according to anteroposterior changes in osteoderm morphology. For the purpose of this work we use “cervical lateral spines” for the those occurring between the skull and the pectoral girdle, “pectoral lateral spines” for those located adjacent to the pectoral girdle, and “thoracal lateral spines” for those situated between the pectoral girdle and the pelvic region (following Ford [9]). Because of the morphological similarity of the Gronau osteoderm with the pectoral spines of *Hylaeosaurus armatus* Mantell, 1833 [10] (see discussion), we decided to apply the anatomical orientation of these elements [11].

The comparison to other Early Cretaceous thyreophorans are based on references as specified in the discussion section as well as on detailed photographies of the holotype specimen (NHM R3775) of *H. armatus*. In order to assess the relationships of the humerus fragment discussed herein, an extensive survey of the references on thyreophoran humerus morphology was undertaken, which shows a systematic and informative distribution of characters with respect to the distal condyli (method, results and references in supplementary information Text S1).

Measurements were taken with a caliper.

### Institutional Abbreviations

DLM, Drilandmuseum, Gronau in Westfalen, Germany, GPMM, Geomuseum of the Westfälische Wilhelms University, Münster in Westfalen, Germany, NHM, Natural History Museum, London, United Kingdom.

## Results

### Systematic paleontology

Ornithischia Seeley, 1888 [12].

Thyreophora Nopcsa, 1915 [13].

Ankylosauria Osborn, 1923 [14].

*Hylaeosaurus* Mantell, 1833 [10].

**Figure 3:**
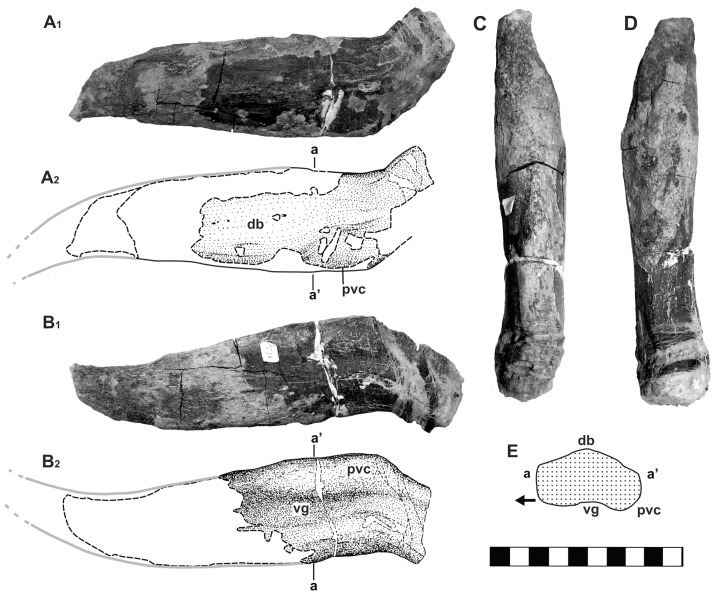
Cervico-pectoral lateral spine of ?*Hylaeosaurus* sp. (DLM 537) from the Bückeberg Formation (early Valanginian) of Gronau in Westfalen, northwestern Germany. A, dorsal view; B, ventral view; C, posterior view; D, anterior view. E, cross-section at a–a′; arrow points anteriorly. White areas in dashed lines: areas with missing substantia compacta; gray line: reconstructed outline. Note that the drawings have been slightly reconstructed to compensate for distortion of the basal part. Abbreviations: db, dorsal bulge; pvc, posteroventral crest; vg, ventral groove. Scale-bar units equal 1 cm.

**Figure 4:**
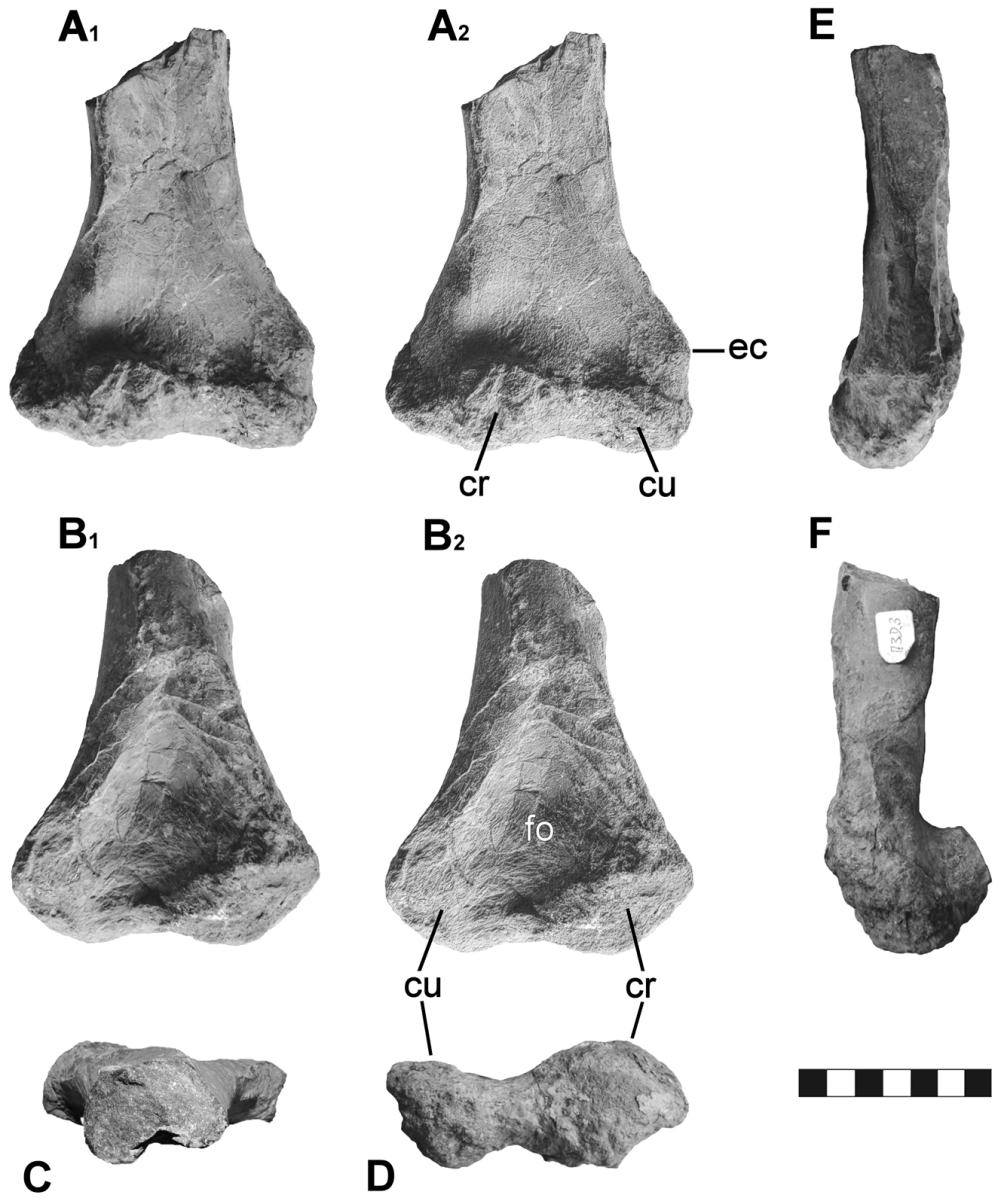
Distal portion of a right humerus of ?*Hylaeosaurus* sp. (GPMM A3D.3) from the Bückeberg Formation (early Valanginian) of Gronau in Westfalen, northwestern Germany. A, anterior view; B, posterior view; C, proximal view; D, distal view; E, medial view; F, lateral view. Figures A2 and B2 have been digitally enhanced. Abbreviations: cr, condylus radialis; cu, condylus ulnaris; ec, entepicondylus; fo, fossa olecrani. Scale-bar units equal 1 cm.

?*Hylaeosaurus* sp.

(Figs. 3–4, Table 1)

**Table 1:** Measurements of the cervico-pectoral lateral spine (DLM 537) and the humerus (GPMM A3D.3) from the early Valanginian of Gronau in Westfalen, northwestern Germany.

Element	Dimensions	Measurements
Lateral spine	Length apicobasal	199*
	Mid-diameter anteroposteriorly	49
	Anteroposterior diameter at lateral end	28
	Maximum anteroposterior diameter	51
	Dorsoventral diameter at preserved apical end	35
	Dorsoventral diameter at preserved basal end	29
	Length of ventral groove	68*
Humerus	Proximodistal length	145*
	Mediolateral width of distal end	103
	Anteroposterior diameter of condylus radialis	35
	Anteroposterior diameter of condylus ulnaris	26

All measurements in millimeters (* marks preserved length of incomplete elements).

#### Material

DLM 537, right cervico-pectoral lateral spine (Fig. 3; Table 1); GPMM A3D.3, distal portion of a right humerus (Fig. 4; Table 1).

#### Stratigraphic Provenance

‘Wealden 6’ (*Pachycytheridea trapezoidalis* ostracod subzone), upper Osterwald Member, Bückeberg Formation, early Valanginian, Early Cretaceous [7], [15], [16], [17].

#### Locality

Gerdemann clay-pit (abandoned), NW of Gronau in Westfalen, Borken district, North Rhine-Westphalia, northwestern Germany.

### Osteological description

#### Cervico-pectoral lateral spine (DLM 537)

The cervico-pectoral spine (Fig. 3) represents most of a slender and pointed osteoderm. Basally it has a subrectangular cross-section, becoming more elliptical apically. The long axis of the cross-section is oriented anteroposteriorly. Its apex and base are missing and the preserved basal-most section is broken and displaced by shearing. Despite this distortion, it is evident that the base tapers basally in an anteroposterior, as well as, in a dorsoventral direction and is anteriorly and weakly ventrally deflected at its base. The preserved section of the osteoderm is curved posteriorly and the sides converge to form the apex. In the basal half of the posterior edge, a blunt, bulging crest (posteroventral crest in Fig. 3 E–G) is formed that thins out apically. Towards the dorsal side, the crest is confluent with the smoothly curved surface. On the ventral surface, the crest is delimited anteriorly by a groove that shallows and widens apically. The dorsal surface inflates dorsally in the mid-section of the osteoderm, forming a broad bulge pierced by a few vascular foramina. Towards the base of the osteoderm this bulge tapers to a narrow ridge. Most of the apical part of the spine is missing the thin substantia compacta, but a partial, 5 cm long core formed by substantia spongiosa is preserved, showing a posterior and slightly ventral curvature of its apex.

#### Humerus (GPMM A3D.3)

The distal portion of a right humerus (Fig. 4) is preserved. The diaphysis and the distal epiphysis are anteroposteriorly compressed, and the maximal constriction of the diaphysis is situated immediately proximal to the epiphysis. The angle formed between the long axis of the diaphysis and a baseline defined by the distal condyli is about 90°. The condylus radialis is larger than the condylus ulnaris, and is distinctly protruding anteriorly in a right angle from the diaphysis in lateral view (Fig. 4F), whereas both protrude distally to the same distance. The condylus ulnaris is accompanied proximomedially by a distinct entepicondylus. The presence of an ectepicondylus cannot be ascertained due to erosion of the bone surface proximolaterally to the condylus radialis. The fossa intercondylaris is shallow and well defined. On the posterior side, above the condyli, a rhomboidal fossa olecrani, is present that reaches proximally to about the middle of the humerus fragment. Anteriorly, a fossa is present that extends from medially, above the condylus ulnaris, to the proximal break.

## Discussion

### Comparisons

The shape and size of the osteoderm is similar to dermal elements known in thyreophoran dinosaurs. Barrett and Maidment [18] note that distinguishing between stegosaurian and ankylosaurian isolated dermal elements must be done with caution, because there is some morphological overlap between these groups. Both are known from the Valanginian through Barremian of Western Europe ([18], [19] for overview). The osteoderm from Gronau differs from typical stegosaur spikes by its longitudinal groove on the ventral side of the basal section, the posteroventral bulging crest and the posteriorly-curved apical section. The elliptical to subrectangular cross-section, which is basally asymmetrical, is also different from the symmetrically ovate to circular cross-section in stegosaur caudal spikes [20], [21], [22]. The spine-shaped plates (‘splates’ sensu Blows [23]) of *Kentrosaurus aethiopicus* Hennig, 1915 [24] from the posterior thoracic region are superficially similar in outline, but the anterior and posterior edges are sharp and blade-like [25] instead of rounded. Additionally, the base of stegosaurian spikes often acutely expands parallel to the body wall to varying degrees. Due to the damage of the base of the Gronau osteoderm, the absence of such an expansion cannot be verified with certainty. However, the basal-most part of the osteoderm is tapering proximally, a condition unknown in spines and spikes with broad basal sections in which the base is normally gently expanded (e.g. [23]).

**Figure 5:**
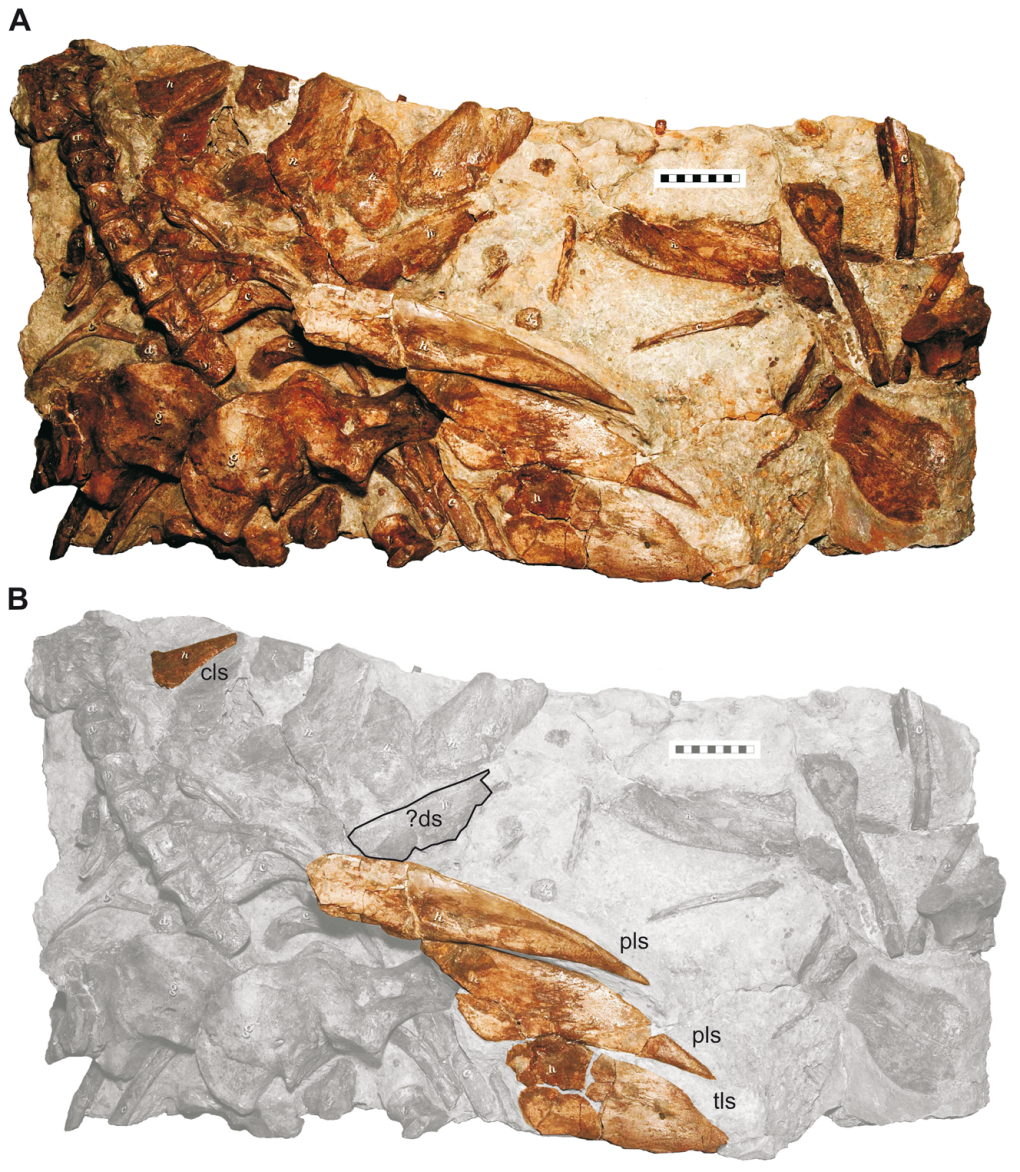
Holotype specimen of *Hylaeosaurus armatus* Mantell, 1833 (NMH R3775) from the Grinstead Clay Formation (late Valanginian) of Tilgate Forest, Sussex, southern England. A, skeleton as preserved; B, purported elements of lateral row of osteoderms highlighted. Abbreviations: cls, cervical lateral spine; ?ds, supposed dorsal spine, considered a pectoral lateral spine by [11] (see text); pls, pectoral lateral spine; tls, thoracal lateral spine. Scale-bar units equal 1 cm. Photo courtesy M. Triebold.

**Figure 6:**
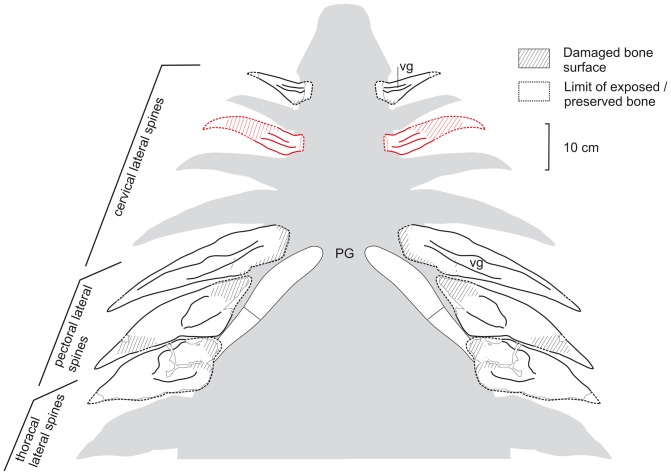
Tentative reconstruction of the lateral cervico-thoracal armor in *Hylaeosaurus armatus.* The reconstruction is shown in ventral aspect, based upon NMH R3775 (holotype, white elements) and added by DLM 537 (red element). No scale intended. **Abbreviations: PG**, pectoral girdle (approx. position, schematic); **vg**, ventral groove.

More similarities are present between the Gronau osteoderm and ankylosaur spines. However, in most taxa lateral and dorsal spines have broad, hollow bases [23], [26], [27], [28], [29] or are basally expanded. Lateral spines may have sharp, longitudinal keels and exhibit a distinct triangular cross-section in some taxa [9]. However, the Gronau spine shows close similarities to the cervical and pectoral spines of at least one taxon, *Hylaeosaurus armatus* Mantell, 1833 [10] from the late Valanginian of southern England. Among the preserved dermal elements [11] of the holotype specimen (NHM R3775 [30], Fig. 5, 6), four spines could be related to the cervical, pectoral, and anterior thoracal lateral series. These include the first spine immediately behind the skull and a series of three spines located near the thoracal vertebral series of the semi-articulated holotype (the latter marked “s′” in [11]: fig. 1b); the posterior of which is incomplete. A fourth spine marked “s′” in [11]: fig. 1b, situated anterior to the others of the thoracal series, does not seem to belong to the lateral row. A picture (courtesy M. Triebold) of this element on a cast of NHM R3775, made with the base of the posteriorly located spine removed, shows a more symmetrical, cone-shaped base of this osteoderm, suggesting its relation to the dorsal series of spines.

The lateral spines show morphological differences between the cervical one and the pectoral and thoracal series. The cervical lateral spine has an inflated, apparently subovate, cross-section and tapers rapidly to a gently posteriorly recurved apex. The shaft of the spine probably extruded in a right angle to the long axis of the animal's body, whereas its most basal part is deflected anteriorly similar to the basal part in DLM 537. It also has a rounded posterobasal crest and a rather steep, flat anterior face. The spines in the pectoral and anterior thoracal lateral series differ mostly from the cervical lateral spine by their dorsoventral flatness which becomes more prominent posteriorly along the series. The two most posterior spines are more similar to triangular plates than to true spines, whereas the anterior thoracal spine has a thicker cross-section. Due to damage, the presence of an anteriorly curved base is not recognizable in the pectoral and anterior thoracal lateral spines. In the pectoral and anterior thoracal series the spines are slanted posteriorly at an angle of about 30°–40° with respect to the transverse axis of the body. However all lateral spines of *H. armatus* as well as DLM 537 share the presence of a ventral longitudinal groove, which is most prominent on the anterior pectoral spine and becomes an increasingly shallow and broad depression on the posterior pectoral and anterior thoracal spines. All lateral spines of *H. armatus* show a posterobasal expansion of the shaft, which has the shape of a bulge-like crest in the anterior cervical spine but flattens to a more wing-like structure in the posterior pectoral and anterior thoracal spines. In addition to this groove, DLM 537 shares with the anterior cervical lateral spine of *H. armatus* an anteroposterior constriction of the posterobasal part of the shaft and a posteroventral bulge-like crest, whereas the shaft is anteroposteriorly wider (dorsoventrally flatter) than in the former. In its overall morphology, DLM 537 would fit well into a transitional series between the anterior cervical and the anterior pectoral spine in *H. armatus* (Fig. 6). This supports a posterior cervical or anterior pectoral position of the element in an animal with similar lateral spines. In order to express the slight uncertainties on the position of the osteoderm we designate it as a cervico-pectoral lateral spine. At present, there is no other ankylosaur known with these distinctive characters of the lateral spines. The genus *Polacanthus* Owen, in Anonymous 1865 [31] from the Hauterivian-Barremian of southern England differs from *Hylaeosaurus* in possessing triangular pectoral spines with expanded bases and sharp anterior and posterior keels [23], [32], [33] and the difference in the morphology between the pectoral spines is considered useful to distinguish between the genera by Barrett and Maidment [18].

Due to the morphological similarity and the close stratigraphical proximity to the English material, the spine from Gronau is therefore tentatively referred to as ?*Hylaeosaurus* sp

As noted previously [4], the humerus likely belongs to an ankylosaurian as well. This assignment is supported by the morphology of the distal condyli, which protrude distally to the same plane, a condition commonly present in ankylosaurs, but unknown in stegosaurs (see supplementary information [Text S1, Fig. S1, Table S1] for detailed evaluation of this character).

Unfortunately, the humerus is not described in *Hylaeosaurus*, although a fragment may be included with the holotype (see Fig.1b in [11]). The Gronau humerus is similar but not identical to the humerus of *Polacanthus foxii* Owen, in Anonymous 1865 [31] ([4]). Its size, compared to corresponding elements of other ankylosaurs [34], indicates that it originates from a small individual, probably in the size range of *H. armatus*, which may be estimated to a total length of 3 to 4 m [35]. The rarity of terrestrial elements in the Gronau reptile fauna and the presence of two ankylosaurian skeletal elements indicative of similar body size give the idea that the material might belong to the same individual some credence; however, this remains conjectural. Given the tentative nature of open nomenclature we nonetheless feel it justified to refer the humerus also to ?*Hylaeosaurus* sp., although a direct comparison of this element with *H. armatus* is not possible at present.

### Record of Thyreophoran Dinosaurs from the Early Cretaceous of Germany

Koken [36] first described two fragmentary caudal vertebral centra from the Berriasian-Valanginian of Duingerwald near Weenzen (Lower Saxony, Northern Germany) that he referred to *Hylaeosaurus* sp.. They were considered lost, but one of them has recently been relocated in the collection of the Niedersächsisches Landesmuseum in Hannover. It is poorly preserved, affected by severe crushing (J. Hornung pers obs.), and its assignment to a thyreophoran is tentative (its referral to *Hylaeosaurus* was already dismissed by [11]). From the lowermost Valanginian (Osterwald Member) of Gretenberg near Sehnde (Lower Saxony) Windolf [37] reported a tooth, a long-bone fragment, a calcaneum and a hoof-like ungual, as belonging to an ankylosaur. This material was not figured and has not been formally described to date, but a recent review of most of the material (the tooth could not be found) from the Roemer-Pelizaeus Museum, Hildesheim and the Geoscience Centre, University of Göttingen, did not reveal any thyreophoran affinities (J. Hornung, unpubl. data).

An ichnological record of ankylosaurs is represented by the ichnospecies *Metatetrapous valdensis* Nopcsa, 1923 [38], known from a single trackway found in 1921 in the Berriasian Obernkirchen Member of the Bückeberg Formation near Bückeburg (Lower Saxony [39]). The trackway is considered lost, but two hypichnial casts of ankylosaurian pedal imprints from the same stratum and area of origin have been identified recently [40]. In conclusion, the osteological material from Gronau and the tracks from Bückeburg represent the only material from Germany unequivocally referable to the Ankylosauria at present.

## Supporting Information

Figure S1 **Assessment of anterodistal protrusion of distal condyli in thyreophoran humeri.** Examples partially reversed to show the same aspect, no scale intended. **A**, Condylus radialis and condylus ulnaris protrude to the same plane, (Ankylosauria: *Niobrarasaurus coleii*, after Carpenter et al. [S51], modified). **B**, Condylus radialis protrudes farther anterodistally than condylus ulnaris (Ankylosauria: *Peloroplites cedrimontanus*, after Carpenter et al. [S33], modified). **C**, Condylus ulnaris protrudes farther anterodistally than condylus radialis (Stegosauria: *Loricatosaurus priscus*, after Galton [S15] [as *Lexovisaurus durobrivensis*], modified). **Abbreviations: cu**, condylus ulnaris; **cr**, condylus radialis; **dp**, crista deltopectoralis.
(TIF)

Table S1 **Character distribution for distal condyli morphology in thyreophoran dinosaurs.** Taxonomy follows Maidment et al. [S10], Carpenter [S11] (Stegosauria), and Thompson et al. [S12] (Ankylosauria), respectively. Only taxa where the humerus is known are included.
(PDF)

Text S1 **Distal humerus morphology in Thyreophora.**
(PDF)
