# Supplementary figures and images for: Ankylosaur Remains from the Early Cretaceous (Valanginian) of Northwestern Germany
Source: PLoS One. 2013 Apr 3;8(4):e60571. doi: 10.1371/journal.pone.0060571 (PMC3616133; doi:10.1371/journal.pone.0060571)

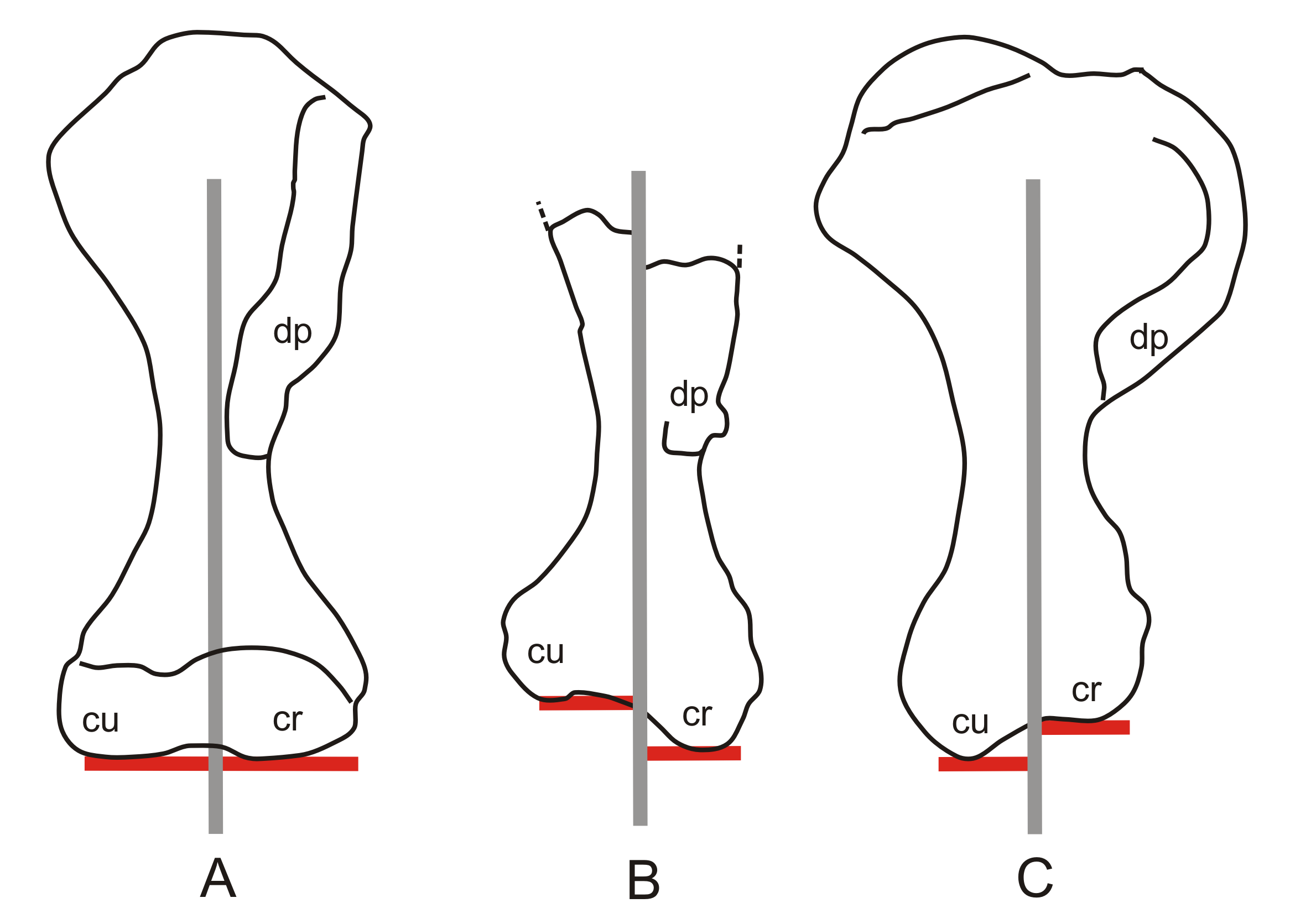

Supplement: Figure S1 — Assessment of anterodistal protrusion of distal condyli in thyreophoran humeri. Examples partially reversed to show the same aspect, no scale intended. A, Condylus radialis and condylus ulnaris protrude to the same plane, (Ankylosauria: Niobrarasaurus coleii, after Carpenter et al. [S51], modified). B, Condylus radialis protrudes farther anterodistally than condylus ulnaris (Ankylosauria: Peloroplites cedrimontanus, after Carpenter et al. [S33], modified). C, Condylus ulnaris protrudes farther anterodistally than condylus radialis (Stegosauria: Loricatosaurus priscus, after Galton [S15] [as Lexovisaurus durobrivensis], modified). Abbreviations: cu, condylus ulnaris; cr, condylus radialis; dp, crista deltopectoralis. (TIF) [file pone.0060571.s001.tif]
