# Supplementary material for: Ankylosaur Remains from the Early Cretaceous (Valanginian) of Northwestern Germany
Source: PLoS One. 2013 Apr 3;8(4):e60571. doi: 10.1371/journal.pone.0060571 (PMC3616133; doi:10.1371/journal.pone.0060571)
Supplement: Table S1 — Character distribution for distal condyli morphology in thyreophoran dinosaurs. Taxonomy follows Maidment et al. [S10], Carpenter [S11] (Stegosauria), and Thompson et al. [S12] (Ankylosauria), respectively. Only taxa where the humerus is known are included. (PDF) [file pone.0060571.s002.pdf]

	Taxon	Condylus ulnaris protruding	Condylus radialis protruding	Condyli at the same plane	Insufficient data	Remarks	Data sources
	„*Chialingosaurus kuani*“ Young, 1959 [S13]	x				=Stegosauridae indet.	[S14]
	Chungkingosaurus sp.	x					[S15]
	Dacentrurus armatus (Owen, 1875) [S16]	x					[S17]
	Gigantspinosaurus sichuanensis Ouyang, 1992 [S18]				x	Information not provided	[S18]
	Hesperosaurus mjosi Carpenter, Miles & Cloward, 2001 [S19]				x	Information not provided	[S11], [S19]
	Huayangosaurus taibaii Dong, Tang & Zhou, 1982 [S20]	x					[S21]
**Stegosauria**	*Kentrosaurus aethiopicus* Hennig, 1915 [S22]	x					[S23]
	Loricatosaurus priscus (Nopcsa, 1911) [S24]	x					[S17]
	Miragaia longicollum Mateus, Maidment & Christiansen, 2009 [S25]	x					[S25]
	Stegosaurus homheni (Dong, 1973) [S26]				x	Information not provided	[S26], [S27]
	Stegosaurus armatus Marsh, 1877 [S28]	x					[S7]
	Tuojiangosaurus multispinus Dong, Li, Zhou & Zhang, 1977 [S29]	x					[S15]
	Ahshislepelta minor Burns & Sullivan, 2011 [S30]				x	Humerus incomplete	[S30]
	Aletopelta coombsi Ford & Kirkland, 2001 [S31]				x	Humerus damaged	[S31]
	Animantarx ramaljonesi Carpenter, Kirkland, Burge & Bird, 1999 [S32]				x	Humerus crushed	[S32]
	Ankylosaurus magniventris Brown, 1908 [S33]			x			[S6]
	Anoplosaurus curtonotus Seeley, 1879 [S34]				x	Humerus incomplete	[S34]
	Cedarpelta bilbeyhallorum Carpenter, Kirkland, Burge & Bird, 2001 [S35]		x				[S36]
	Crichtonsaurus bohlini Dong, 2002 [S37]				x	Information not provided	[S37]
	„*Crichtonsaurus*“ *benxiensis* Lü, Ji, Gao & Li, 2007 [S38]		x				[S38]
	Edmontonia rugosidens (Gilmore, 1930) [S39]			?		Information not provided	[S40]
	Euoplocephalus tutus Lambe, 1902 [S41]			x			[S1]
	Gargoyleosaurus parkpinorum Carpenter, Miles & Cloward, 1998 [S42]				x	Humerus crushed	[S43]
	Gastonia burgei Kirkland, 1998 [S44]			x			[S45]
	Gobisaurus domoculus Vickaryous, Russell, Currie & Zhao, 2001 [S46]				x	Information not provided	[S46]
	Hoplitosaurus marshi (Lucas, 1901) [S47]			x			[S48]
	Hungarosaurus tormai Ösi, 2005 [S49]	x					[S2]
	Liaoningosaurus paradoxus Xu, Wang & You, 2001 [S50]				x	Early ontogenetic stage	[S50]
**Ankylosauria**	*Minmi paravertebra* Molnar, 1980 [S51]			x			[S52]
	Niobrarasaurus coleii (Mehl, 1936) [S53]			x			[S54]
	Panoplosaurus mirus Lambe, 1919 [S55]			x			[S54]
	Peloroplites cedrimontanus Carpenter, Bartlett, Bird & Barrick, 2008 [S36]		x				[S36]
	Pinacosaurus grangeri Gilmore, 1933 [S56]			x			[S3]
	Pinacosaurus mephistocephalus Godefroit, Pereda-Suberbiola, Li & Dong, 1999 [S57]				x	Information not provided	[S57]
	Polacanthus foxii Owen, in Anonymous 1865 [S58]			x		Referred isolated humerus	[S48]
	Polacanthus rudgwickensis Blows, 1996 [S59]				x	Humerus fragmentary	[S59]
	Saichania chulsanensis Maryanska, 1977 [S3]	x					[S3]
	Sauropelta edwardsorum Ostrom, 1970 [S60]			x			[S60]
	Scolosaurus cutleri Nopcsa, 1928 [S4]				x	Humerus incomplete	[S5]
	Shamosaurus scutatus Tumanova, 1983 [S61]				x	Information not provided	[S61]
	Shanxia tianzhenensis Barrett, You, Upchurch & Burton, 1998 [S62]			x			[S62]
	Struthiosaurus austriacus Bunzel, 1871 [S63]				x	Humerus incomplete	[S64]
	Talarurus plicatospineus Maleev, 1952 [S65]			x			[S65]
	Texasetes pleurohalio Coombs, 1995 [S66]			?		Humeri incomplete	[S67]
	Tianzhenosaurus youngi Pang & Cheng, 1998 [S68]			x			[S68]
	Zhongyuansaurus luoyangensis Li, Lü, Zhang, Jia, Hu, Zhang, Wu & Ji, 2007 [S69]				x	Information not provided	[S69]
