# Supplementary material for: Ankylosaur Remains from the Early Cretaceous (Valanginian) of Northwestern Germany
Source: PLoS One. 2013 Apr 3;8(4):e60571. doi: 10.1371/journal.pone.0060571 (PMC3616133; doi:10.1371/journal.pone.0060571)
Supplement: Text S1 — Distal humerus morphology in Thyreophora. (PDF) [file pone.0060571.s003.pdf]

SUPPLEMENTARY INFORMATION

S1 Distal humerus morphology in Thyreophora

Methods

As in most dinosaurs, the morphology of the distal part of the humerus is variable among taxa (and, to a lesser degree between individuals, e.g. Penkalski [S1]), especially with respect to the relative size and morphology of the distal condyli. However, the systematic distribution of these differences has not been considered yet for the purpose of assigning distal humeri of thyreophorans to distinct subclades of the group.

Data mining across a wide range of thyreophorans shows that the relative proximodistal position of the distal condyli is promising for a systematic distribution. We used references for humeri of all available stegosaurian and ankylosaurian taxa from the literature. To establish and qualify the position of the distal condyli, we compared (in anterior aspect of the humerus) the distance between the points, where a horizontal line, tangential to the distalmost point of each condylus intersect a line parallel to the diaphysis in a right angle (Fig. S1-1). If these lines meet in the same location, the condyli are considered equal in proximodistal expansion (Fig. S1-1A); if the line tangentially to the condylus ulnaris intersects above that one tangentially to the condylus radialis, the latter is considered to protrude (antero)distally (Fig. S1-1B) and vice versa (Fig. S1-1C). In fact, the protrusion of the condyli has also an anterior component, which is obscured when seen from the posterior side of the humerus, which then results in an underestimation of the condylar inequality. Due to the variability of source quality we choose to present the results in a qualitative form (Tab. S1-1).

Results

A comparison of the distal humerus morphology shows clear differences between stegosaurs and ankylosaurs. In stegosaurs uniformly the condylus ulnaris is anterodistally protruding. The protrusion is generally well marked, at least in adult specimens.

Contrary, most ankylosaurian taxa share a condition where both condyli are located at the same plane, only in *Cedarpelta bilbeyhallorum*, *„Crichtonsaurus“ benxiensis*, and *Peloroplites cedrimontanus* the condylus radialis is protruding anterodistally. Among the surveyed taxa only two exceptions were recognized: In *Hungarosaurus tormai* the condylus ulnaris is more protruding distally than the condylus radialis, though the latter is larger in overall size (Ösi & Makádi [S2]). In *Saichania chulsanensis* the condylus ulnaris is more massive and protruding distally than the condylus radialis (Maryanska [S3]: pl. 35, fig. 1b). In the latter taxon, as well as in an indetermined ankylosaurine humerus (possibly belonging to *Scolosaurus cutleri* Nopcsa, 1928 [S4], see Penkalski & Blows [S5]: fig. 6a), the condylus radialis is reduced in size and prominence but the laterodistal margin of the humerus is formed by a flange-like, large ectepicondylus.

In the few instances where a series of individuals of the same species is described (*Euoplocephalus tutus*: Penkalski [S1]; *Ankylosaurus magniventris*: Carpenter [S6]; *Stegosaurus armatus*: Gilmore [S7], Galton [S8]), the inequality in condylar protrusion can vary between individuals. However, the current record indicates that if a protrusion is recognizable, within the same species, always the same condylus protrudes. The morphology of the distal condyles may be strongly influenced by the ontogenetic stage of the individual due to increasing ossification of the epiphyseal cartilage with increasing individual age (e. g. Holliday et al. [S9]).

According to this dataset, an anterodistally protruding condylus ulnaris is the common condition in Stegosauria and occurs very rarely in Ankylosauria. An equality of distal protrusion of both condyli is the common condition in Ankylosauria with some taxa showing an anterodistal protrusion of the condylus radialis. The latter conditions are entirely unknown in Stegosauria, though the inequality in distal condylar protrusion is less pronounced in some juvenile individuals of *Stegosaurus armatus* (see Galton [S8]).

Based upon these results, we feel it justified to use the orientation of the distal condyli in thyreophorans to assign distal humeri to either of the subclades, further supporting a referral of GPMM A3D.3 to the Ankylosauria.

References for Supporting Information Text and Table S1

[S1] Penkalski PG (2001) Variation in specimens referred to *Euoplocephalus tutus*. In: Carpenter K, editor. The armored dinosaurs. Bloomington: Indiana University Press. pp. 261-298.

[S2] Ösi A, Makádi L (2009) New remains of *Hungarosaurus tormai* (Ankylosauria, Dinosauria) from the Upper Cretaceous of Hungary: skeletal reconstruction and body mass estimation. Paläontologische Zeitschrift 83: 227-245.

[S3] Maryanska T (1977) Ankylosauridae (Dinosauria) from Mongolia. Palaeontologia Polonica 37: 85-151.

[S4] Nopcsa F (1928) Paleontological notes on reptiles. Geologica Hungarica, Series Palaeontologica 1: 51-74.

[S5] Penkalski PG, Blows WT (2013) *Scolosaurus cutleri* (Ornithischia: Ankylosauria) from the Upper Cretaceous Dinosaur Park Formation of Alberta, Canada. Can J Earth Sci 38 (advance online publication) doi: 10.1139/cjes-2012-0098.

[S6] Carpenter K (2004) Redescription of *Ankylosaurus magniventris* Brown 1908 (Ankylosauridae) from the Upper Cretaceous of the Western Interior of North America. Can J Earth Sci 41: 961–986.

[S7] Gilmore CW (1914) Osteology of the armored Dinosauria in the United States National Museum, with special reference to the genus *Stegosaurus.* United States National Museum Bulletin 89: 1–136.

[S8] Galton P (1982) Juveniles of the stegosaurian dinosaur *Stegosaurus* from the Upper Jurassic of North America. Journal of Vertebrate Paleontology 2: 47–62.

[S9] Holliday CM, Ridgely RC, Sedlmayr JC, Witmer LM (2010) Cartilaginous Epiphyses in Extant Archosaurs and Their Implications for Reconstructing Limb Function in Dinosaurs. PLoS ONE 5(9): e13120. doi:10.1371/journal.pone.0013120

[S10] Maidment SCR, Norman DB, Barrett PM, Upchurch P (2008) Systematics and phylogeny of Stegosauria (Dinosauria: Ornithischia). Journal of Systematic Palaeontology 6: 364-407.

[S11] Carpenter K (2010) Species concept in North American stegosaurs. Swiss Journal of Geosciences 103:155-162.

[S12] Thompson RS, Parish JC, Maidment SCR, Barrett PM (2012) Phylogeny of the ankylosaurian dinosaurs (Ornithischia: Thyreophora). Journal of Systematic Palaeontology 10: 301-312.

[S13] Young CC (1959) On a new Stegosauria from Szechuan, China. Vertebrata PalAsiatica 3: 1-8.

[S14] Maidment SC, Wei G (2006) A review of the Late Jurassic stegosaurs (Dinosauria, Stegosauria) from the People's Republic of China. Geol Mag 143: 621–634.

[S15] Dong Z, Zhou SW, Zhang YH (1983) The dinosaurian remains from Sichuan Basin, Chinas (in Chinese). Palaeontologica Sinica, New Series C 162: 1–136.

[S16] Owen R (1875) Monographs on the fossil Reptilia of the Mesozoic formations. Part II. (Genera *Bothriospondylus, Cetiosaurus, Omosaurus).* The Palaeontographical Society, London 1875: 15-93.

[S17] Galton P (1985) British plated dinosaurs (Ornithischia, Stegosauridae). Journal of Vertebrate Paleontology 5: 211–254

[S18] Ouyang H (1992) Discovery of *Gigantspinosaurus sichanensis* and its scapular spine orientation (in Chinese). Abstracts and Summaries for Youth *Academic* Symposium on New Discoveries and Ideas in Stratigraphic Paleontology null: 47–49.

[S19] Carpenter K, Miles C, Cloward K (2001) New primitive stegosaur from the Morrison Formation, Wyoming. In: Carpenter K, editor. The armored dinosaurs. Bloomington: Indiana University Press. pp. 55-75.

[S20] Dong Z, Tang Z, Zhou SW (1982) Note on the new Mid-Jurassic stegosaur from Sichuan Basin, China (in Chinese). Vertebrata PalAsiatica 20: 83-87.

[S21] Zhou S (1984) The Middle Jurassic Dinosaur Fauna from Dashanpu, Zigong, Sichuan. Vol. II: Stegosaurs (in Chinese). Sichuan: Sichuan Scientific and Technological Publishing House, 52 pp..

[S22] Hennig E (1915) *Kentrosaurus aethiopicus* der Stegosauride des Tendaguru. Sitzungsberichte der Gesellschaft naturforschender Freunde zu Berlin 1915: 219-247.

[S23] Hennig E (1925) *Kentrurosaurus aethiopicus* - Die Stegosaurier-Funde vom Tendaguru, Deutsch-Ostafrika. Palaeontographica Suppl 7:101-254.

[S24] Nopcsa F (1911) Notes on British dinosaurs. Part IV: *Stegosaurus priscus*, sp. nov.. Geol Mag 5: 109-115.

[S25] Mateus O, Maidment SCR, Christiansen NA (2009) A new long-necked 'sauropod-mimic' stegosaur and the evolution of the plated dinosaurs. Proc Biol Sci 276: 1815-1821.

[S26] Dong Z (1973) Dinosaurs from Wuerho (in Chinese). Reports of Paleontological Expedition to Sinkiang (II): Pterosaurian Fauna from Wuerho, Sinkiang. Memoirs of the Institute of Vertebrate Paleontology and Paleoanthropology, Academia Sinica 11:45-52.

[S27] Dong Z (1990) Stegosaurs of Asia. Carpenter K, Currie PJ, editors. Dinosaur Systematics: Cambridge University Press: 255–268.

[S28] Marsh OC (1877) A new order of extinct Reptilia (Stegosauria) from the Jurassic of the Rocky Mountains. Am J Sci 14: 513-514.

[S29] Dong Z, Li X, Zhou S, Zhang Y (1977) On the stegosaurian remains from Zigong (Tzekung), Szechuan province (in Chinese). Vertebrata PalAsiatica 15:307-312.

[S30] Burns ME, Sullivan RM (2011) A new ankylosaurid from the Upper Cretaceous Kirtland Formation, San Juan Basin, with comments on the diversity of ankylosaurids in New Mexico. In: Sullivan RM, Lucas SG, Spielmann JA. Fossil Record 3. New Mexico Museum of Natural History and Science Bulletin 53: 169-178.

[S31] Ford T, Kirkland JI (2001) Carlsbad ankylosaur (Ornithischia, Ankylosauria): An ankylosaurid and not a nodosaurid. In: Carpenter K, editor. The armored dinosaurs. Bloomington: Indiana University Press. pp. 239-260.

[S32] Carpenter K, Kirkland JI, Burge D, Bird J (1999) Ankylosaurs (Dinosauria: Ornithischia) of the Cedar Mountain Formation, Utah, and their stratigraphic distribution. In: Gillette D,

editor. Vertebrate Paleontology in Utah. Utah Geological Survey Miscellaneous Publication 99-1: 243-251.

[S33] Brown B (1908) The Ankylosauridae, a new family of armored dinosaurs from the Upper Cretaceous. Bull Am Mus Nat Hist 24: 187-201.

[S34] Seeley HG (1879) On the Dinosauria of the Cambridge Greensand. Quart J Geol Soc London 35: 591-636.

[S35] Carpenter K, Kirkland JI, Burge D, Bird J (2001) Disarticulated skull of a new primitive ankylosaurid from the Lower Cretaceous of Eastern Utah. In: Carpenter K, editor. The armored dinosaurs. Bloomington: Indiana University Press. pp. 211-238.

[S36] Carpenter K, Bartlett J, Bird J, Barrick R (2008) Ankylosaurs from the Price River Quarries, Cedar Mountain Formation, (Lower Cretaceous), East-Central Utah. Journal of Vertebrate Paleontology 28: 1089–1101.

[S37] Dong Z (2002) A new armored dinosaur (Ankylosauria) from Beipiao Basin, Liaoning Province, northeastern China (in Chinese). Vertebrata PalAsiatica 40: 276–285.

[S38] Lü J, Ji Q, Gao Y, Li Z (2007) A new species of the ankylosaurid dinosaur *Crichtonsaurus* (Ankylosauridae: Ankylosauria) from the Cretaceous of Liaoning Province, China. Acta Geologica Sinica 81: 883–897.

[S39] Gilmore CW (1930) On dinosaurian reptiles from the Two Medicine Formation of Montana. Proceedings of the United States National Museum 77: 1–39.

[S40] Russell LS (1940) *Edmontonia rugosidens* (Gilmore), an armored dinosaur from the Belly River Series of Alberta. University of Toronto Studies. Geology Series 43: 3-27.

[S41] Lambe LM (1902) New genera and species from the Belly River Series (mid-Cretaceous). Geological Survey of Canada Contributions to Canadian Palaeontology 3: 25-81.

[S42] Carpenter K, Miles C, Cloward K (1998) Skull of a Jurassic ankylosaur (Dinosauria). Nature 393: 782–783.

[S43] Kilbourne B, Carpenter K (2005) Redescription of *Gargoyleosaurus parkpinorum*, a polacanthid ankylosaur from the Upper Jurassic of Albany County, Wyoming. Neues Jahrb Geol Palaontol Abh 237: 111-160.

[S44] Kirkland JI (1998) A polacanthine ankylosaur (Ornithischia: Dinosauria) from the Early Cretaceous (Barremian) of Eastern Utah. In: Lucas SG, Kirkland JI, Estep JW, editors. Lower and Middle Cretaceous Terrestrial Ecosystems. New Mexico Museum of Natural History and Science Bulletin 14: 271–282.

[S45] Gaston RW, Schellenbach J, Kirkland JI (2001) Mounted skeleton of the polacanthine ankylosaur *Gastonia burgei*. In: Carpenter K, editor. The armored dinosaurs. Bloomington: Indiana University Press. pp. 386-398.

[S46] Vickaryous M, Russell AP, Currie PJ, Zhao X (2001) A new ankylosaurid (Dinosauria: Ankylosauria) from the Lower Cretaceous of China, with comments on ankylosaurian relationships. Can J Earth Sci 38: 1767–1780.

[S47] Lucas FA (1901) A new dinosaur, *Stegosaurus marshi,* from the Lower Cretaceous of South Dakota. Proceedings of the United States National Museum 23: 591-592.

[S48] Pereda-Suberbiola J (1994) *Polacanthus* (Ornithischia, Ankylosauria), a transatlantic armoured dinosaur from the Early Cretaceous of Europe and North America. Palaeontographica A 232: 133–159.

[S49] Ösi A (2005) *Hungarosaurus tormai*, a new ankylosaur (Dinosauria) from the Upper Cretaceous of Hungary. Journal of Vertebrate Paleontology 25: 370-383.

[S50] Xu X, Wang XL, You HL (2001) A juvenile ankylosaur from China. Naturwissenschaften 88: 297-300.

[S51] Molnar RW (1980) An ankylosaur (Ornithischia, Reptilia) from the Lower Cretaceous of southern Queensland. Mem Queensl Mus 20: 77-87.

[S52] Molnar RW (1996) Preliminary report of a new ankylosaur from the Early Cretaceous of Queensland, Australia. Mem Queensl Mus 45: 653-668.

[S53] Mehl MC (1936) *Hierosaurus coleii*: a new aquatic dinosaur from the Niobrara Cretaceous of Kansas. Denison University Bulletin 31: 1-20.

[S54] Carpenter K, Dikes D, Weishampel DB (1995) The dinosaurs of the Niobrara Chalk Formation (Upper Cretaceous, Kansas). Journal of Vertebrate Paleontology 15: 275-297.

[S55] Lambe LM (1919) Description of a new genus and species (*Panoplosaurus mirus*) of an armoured dinosaur from the Belly River Beds of Alberta. Trans R Soc Can, series 3 13: 39-50.

[S56] Gilmore CW (1933) Two new dinosaurian reptiles from Mongolia with notes on some fragmentary specimens. Am Mus Novit 679: 1-20.

[S57] Godefroit P, Pereda-Suberbiola X, Li H, Dong Z (1999) A new species of the ankylosaurid dinosaur *Pinacosaurus* from the Late Cretaceous of Inner Mongolia (P. R. China). Bulletin de l'Institut Royal des Sciences Naturelles de Belgique, Sciences de la Terre 69 (suppl): 17-36

[S58] Anonymus (1865) A new Wealden dragon. Order, Sauria; Family, Dinosaurian; Genus, *Polacanthus*; Species, foxii. Illustrated London News 47: 270.

[S59] Blows WT (1996) A new species of *Polacanthus* (Ornithischia; Ankylosauria) from the Lower Cretaceous of Sussex, England. Geol Mag 133: 671-682.

[S60] Ostrom JH (1970) Stratigraphy and Paleontology of the Cloverly Formation (Lower Cretaceous) of the Bighorn Basin Area, Wyoming and Montana. Bull Peabody Mus Nat Hist 35: 1-355.

[S61] Tumanova TA (1983) The first ankylosaurs from the Lower Cretaceous of Mongolia (in Russian). In: Tatarinov LP, Barsbold R, Vorobyeva E, Luvsandanzan B, Trofimov BA, Reshetov YA, Shishkin MA, editors. Fossil Reptiles of Mongolia. Trudy Sovmestnaya Sovetsko-Mongol'skaya Paleontologicheskaya Ekspeditsiya 24:110-118.

[S62] Barrett PM, You H, Upchurch P, Burton AC (1998) A new ankylosaurian dinosaur (Ornithischia: Ankylosauria) from the Upper Cretaceous of Shanxi Province, People's

Republic of China. Journal of Vertebrate Paleontology 18: 376–384.

[S63] Bunzel E (1871) Die Reptilfauna der Gosau-Formation in der Neuen Welt bei Wiener-Neustadt. Abhandlungen der Kaiserlich-Königlichen Geologischen Reichsanstalt 5: 1-19.

[S64] Pereda-Suberiola X, Galton P (2001) Reappraisal of the nodosaurid ankylosaur *Struthiosaurus austriacus* Bunzel from the Upper Cretaceous Gosau Beds of Austria. In: Carpenter K, editor. The armored dinosaurs. Bloomington: Indiana University Press. pp. 173-210.

[S65] Maleev EA (1952) A new ankylosaur from the Upper Cretaceous of Mongolia (in Russian). Doklady Akademii Nauk SSSR 87: 273-276.

[S66] Coombs WP (1995) A nodosaurid ankylosaur (Dinosauria: Ornithischia) from the Lower Cretaceous of Texas. Journal of Vertebrate Paleontology 15: 298-312.

[S67] Carpenter K, Kirkland JI (1998) Review of Lower and Middle Cretaceous ankylosaurs from North America. In: Lucas SG, Kirkland JI, Estep JW, editors. Lower and Middle Cretaceous Terrestrial Ecosystems. New Mexico Museum of Natural History and Science Bulletin 14: 249-270.

[S68] Pang Q, Cheng Z (1998) A new ankylosaur of Late Cretaceous from Tianzhen, Shanxi. Progress in Natural Science 8:326-334.

[S69] Li X, Lü J, Zhang X, Jia S, Hu W, Zhang J, Wu Y, Ji Q (2007) A new nodosaurid dinosaur fossil from the Cretaceous Period of Ruyang, Henan (in Chinese). Acta Geologica Sinica 81: 433-438.
